# Comparisons of Genetic and Clinical Findings in Patients with Syndromic to Non-Syndromic Familial Exudative Vitreoretinopathy

**DOI:** 10.3390/ijms27083348

**Published:** 2026-04-08

**Authors:** Sho Naruse, Takaaki Hayashi, Tomoko Tsukahara-Kawamura, Itsuka Matsushita, Tatsuo Nagata, Sachiko Nishina, Takao Endo, Shunji Kusaka, Hiroyuki Kondo

**Affiliations:** 1 Department of Ophthalmology, University of Occupational and Environmental Health, Japan, Kitakyushu 807-8555, Japan; sho-naruse@med.uoeh-u.ac.jp (S.N.); i-matsushita@med.uoeh-u.ac.jp (I.M.); 941067@med.uoeh-u.ac.jp (T.N.); 2 Department of Ophthalmology, The Jikei University School of Medicine, Tokyo 105-8461, Japan; takaaki@amy.hi-ho.ne.jp; 3 Department of Ophthalmology, Fukuoka University School of Medicine, Fukuoka 814-0180, Japan; ttsukahara@adm.fukuoka-u.ac.jp; 4 Division of Ophthalmology, National Center for Child Health and Development, Tokyo 157-8535, Japan; nishina-s@ncchd.go.jp; 5 Department of Ophthalmology, Osaka Women’s and Children’s Hospital, Izumi 594-1101, Japan; mm1016et@gmail.com; 6 Department of Ophthalmology, Kindai University Faculty of Medicine, Sakai 590-0197, Japan; skusaka@gmail.com

**Keywords:** familial exudative vitreoretinopathy, FEVR, syndromic FEVR, *CTNNB1*, *DOCK6*, *KIF11*, *NDP*

## Abstract

To compare the genetic causes, prevalence, and clinical characteristics of syndromic and non-syndromic familial exudative vitreoretinopathy (FEVR). A total of 281 patients with FEVR who underwent clinical and genetic evaluation at five ophthalmological institutions in Japan between 2010 and 2023 were included. Whole-exome sequencing, Sanger sequencing, or karyotype analysis was performed using blood samples from probands and available family members. Clinical characteristics of FEVR probands were assessed according to the presence or absence of systemic abnormalities. Among the 281 FEVR probands, 42 (15%) had syndromic FEVR and 239 (85%) had non-syndromic FEVR. Syndromic FEVR was more frequently diagnosed during infancy (95% vs. 57%, *p* < 0.0001) and occurred more often in sporadic cases (69% vs. 50%, *p* = 0.028). Variants in Norrin/β-catenin signaling genes were less common in syndromic FEVR (29% vs. 54%, *p* = 0.0026), whereas symmetrical retinal severity was more frequently observed (67% vs. 39%, *p* = 0.001). Sex distribution did not differ between groups. Pathogenic variants were identified in 71% of syndromic cases, most commonly in *KIF11*, *NDP*, *CTNNB1*, *DOCK6*, *TSPAN12*, and *LRP5*. Syndromic FEVR exhibits distinct and heterogeneous genetic and clinical features compared with non-syndromic FEVR. Genotype–phenotype characterization may enable earlier diagnosis.

## 1. Introduction

Familial exudative vitreoretinopathy (FEVR) is an inherited disorder characterized by vascular abnormalities in the peripheral retina [1]. The retinal features of FEVR include peripheral retinal avascularization, retinal exudation, neovascularization, vitreous hemorrhages, falciform retinal folds, and retinal detachments (RDs) [2]. FEVR is characterized by distinctive retinal vascular features, such as V-shaped vascular notches and brushy vascular ends, and its clinical diagnosis can often be made based on these retinal findings alone [3]. FEVR is associated with various types of RDs, including tractional and rhegmatogenous forms, with tractional RD being the most representative [1].

FEVR is a genetically heterogeneous disorder, and the most prevalent genes causing FEVR include *LRP5*, *FZD4*, *TSPAN12*, and *NDP*, which that encode the proteins consisting of the Norrin/β-catenin signaling pathway that controls a transcriptional program that regulates endothelial growth in the retinal vasculature [4]. Mutations in the autosomal genes (*LRP5*, *FZD4* and *TSPAN12*) cause autosomal dominant (or recessive) FEVR, while mutations in the *NDP* gene cause X-linked recessive FEVR [2]. The genotype and phenotype relationship in FEVR is additionally complex. It was originally not linked to systemic anomalies [1]; however, a variety of syndromes that linked to FEVR have been reported [5]. For example, mutations in the *NDP* gene cause Norrie disease, which is an X-linked recessive disorder presenting congenital retinal detachments associated with hearing impairment and intellectual disability [6]. Mutations in the *LRP5* gene can cause osteoporosis pseudoglioma syndrome (OPPG), which is an autosomal recessive disorder presenting systemic features of osteoporosis in addition to retinal detachments [7]. The *CTNNB1* gene, which encodes β-catenin, has recently been reported to be associated with both syndromic and non-syndromic FEVR [8,9]. The *KIF11* gene is not part of the Norrin/β-catenin pathway but causes microcephaly chorioretinopathy syndrome and shows phenotypic overlap with FEVR [10,11]. The *DOCK6* gene, which is responsible for Adams–Oliver syndrome, is reported to be attributed to FEVR with microcephaly [12]. Among these causative genes, certain mutations are associated with characteristic retinal features that may suggest syndromic FEVR; for example, chorioretinopathy is strongly associated with mutations in the *KIF11* gene. However, no specific retinal features have been consistently associated with syndromic FEVR.

However, not all systemic abnormalities are related to FEVR. Some patients may coincidentally carry pathogenic variants for other Mendelian disorders, resulting in a complex phenotype. It is often difficult to determine whether particular systemic findings are truly associated with FEVR. Therefore, we defined syndromic FEVR as FEVR accompanied by systemic abnormalities, after excluding known non-FEVR syndromes at the initial clinical evaluation. In this study, syndromic FEVR refers to patients with FEVR who present with extraocular signs or systemic features, in contrast to non-syndromic FEVR, which is limited to ocular manifestations.

The contribution of syndromic FEVR within the overall FEVR spectrum, including its prevalence and clinical characteristics, remains unclear. This study aimed to assess the prevalence of syndromic FEVR and compare its genetical and clinical features with those of non-syndromic FEVR. We previously reported the clinical characteristics of FEVR with and without pathogenic variants in Norrin/β-catenin pathway genes (*LRP5*, *FZD4*, *TSPAN12*, and *NDP*) in a cohort of 281 Japanese patients, and partially described findings associated with *KIF11* [13,14]. In the present study, we further evaluated phenotype–genotype relationships of genes previously unreported in our cohort, including *CTNNB1* and *DOCK6*.

## 2. Results

### 2.1. Characteristics of Syndromic Versus Non-Syndromic FEVR Patients

Of the 281 FEVR probands, 42 (15%) were classified as syndromic FEVR and 239 (85%) with non-syndromic FEVR. In the syndromic FEVR group (*n* = 42), 23 probands were males and 19 were females, and in the non-syndromic FEVR probands (*n* = 239), 157 were males and 82 were females (Table 1). The differences in the sex distribution between the syndromic (55%) and non-syndromic FEVR (66%) groups was not significant (*p* = 0.2200). Thirteen of the syndromic FEVR probands (31%: 13/42) were familial and 29 (69%: 29/42) were sporadic. The sporadic cases were observed significantly more frequently in the syndromic group than in the non-syndromic FEVR group (*p* = 0.0280). Forty probands (95%: 40/42) in the syndromic group were diagnosed in infancy and two (5%: 2/42) as juveniles or adults. The infant cases were observed significantly more frequently in the syndromic (95%: 40/42) than non-syndromic (57%: 136/239) FEVR groups (*p* < 0.0001). Pathogenic variants in the major Norrin/β-catenin genes, i.e., *LRP5*, *FZD4*, *TSPAN12* and *NDP*, were identified in 12 individuals (29%) in the syndromic FEVR cases, including two with chromosomal rearrangements of the *TSPAN12* gene (Table 2). The Norrin/β-catenin signaling-related gene variants for non-syndromic FEVR were listed in a previous report [13]. Variants of these genes were detected significantly less frequently in the syndromic (29%: 12/42) than non-syndromic (54%: 129/239) FEVR eyes (*p* = 0.0026). Eyes at the same stage bilaterally were found more frequently in the syndromic (67%: 28/42) than the non-syndromic (39%: 94/239) FEVR groups (*p* = 0.0012). Because 95% of syndromic FEVR cases were of the infantile type, we compared interocular symmetry only among patients with the infantile type. Interocular symmetry was more frequent in syndromic FEVR than in non-syndromic FEVR (28/40 [70%] vs. 50/136 [37%], *p* = 0.0003; Table 3).

**Table 1:** Demographic characteristics between probands with syndromic and non-syndromic familial exudative vitreoretinopathy (FEVR).

	Syndromic *n* = 42	Non-Syndromic *n* = 239	*p*
Male	23 (55%)	157 (66%)	
Female	19 (45%)	82 (34%)	0.2200
Familial	13 (31%)	120 (50%)	
Sporadic	29 (69%)	119 (50%)	0.0280
Infantile case	40 (95%)	136 (57%)	
Juvenile or adult case	2 (5%)	103 (43%)	<0.0001
Norrin/β variants positive	12 (29%)	129 (54%)	
Norrin/β variants negative	30 (71%)	110 (46%)	0.0026
Asymmetry	14 (33%)	145 (61%)	
Symmetry	28 (67%)	94 (39%)	0.0012
Stage of more severe eyes			
Stage 1	2 (5%)	36 (15%)	
Stage 2	1 (2%)	11 (5%)	
Stage 3	6 (14%)	35 (15%)	
Stage 4	19 (45%)	82 (34%)	
Stage 5	14 (33%)	33 (14%)	
Stage R	0 (0%)	42 (18%)	0.0013

**Table 2:** Clinical characteristics and genotypes of probands with syndromic FEVR.

Case Number	Sex	Age at Diagnosis	Gene	Nucleotide or Chromosome Change	Protein Change	Familial/Sporadic (Mode of Inheritance)	Stage RE/LE	Systemic Changes and Comment (Affected Family Members)	Reference
1	M	4/12	*KIF11*	c.704C>G	p.S235C	Sporadic (de novo)	4/4	MC, ID, FD	[14]
2	F	6	*KIF11*	c.2842dupC	p.L948fxPfs*2	Sporadic	3/3	MC, ID, FD	This study
3	M	5/12	*KIF11*	c.868C>T	p.Q290*	Familial (AD)	4/4	MC, ID, FD, (affected: mother, maternal aunt)	[14]
4	F	1/12	*KIF11*	c.1159C>T	p.R387*	Familial (AD)	4/4	MC, ID, FD, (affected: 2 brothers, mother, maternal grandfather)	[14]
5	F	1	*KIF11*	c.2777delC	p.T926Nfs*14	Sporadic (de novo)	4/4	MC	[14]
6	M	3/12	*KIF11*	c.1159C>T	p.R387*	Sporadic (de novo)	4/4	MC, ID, FD	[14]
7	F	8/12	*KIF11*	c.2541dupA	p.L848Ifs*9	Sporadic (de novo)	5/5	MC	[14]
8	F	5/12	*KIF11*	Exons 1–21del		Sporadic (de novo)	5/4	MC, ID, FD	[14]
9	F	6/12	*KIF11*	c.1736_1737insATA	p.D579delinsEY	Sporadic (de novo)	4/1	MC	[14]
10	F	1	*KIF11*	Exon 1del		Familial (AD)	4/5	MC, ID, FD, (affected: mother)	[14]
11	F	1	*KIF11*	c.2267+1G>C		Familial (AD)	4/4	MC, ID, (affected: father, brother)	[14]
12	M	6/12	*KIF11*	c.370G>T	p.E124*	Sporadic (de novo)	4/4	MC	This study
13	M	1/12	*NDP*	c.290G>C	p.R97P	Familial (XL)	5/5	ID	[13]
14	M	1	*NDP*	c.175-1G>A		Sporadic	5/5	ID	[13]
15	M	0 ^&^	*NDP*	c.334_340delGGGGGCA	p.G112Cfs*148	Sporadic	5/5	ID	[13]
16	M	1/12	*NDP*	c.376T>G	p.C126G	Sporadic	5/5	ID	[13]
17	M	3/12	*NDP*	c.194G>A	p.C65Y	Sporadic	5/5	ID	[13]
18	M	0 ^&^	*NDP*	c.11_12del	p.H4Rfs*21	Familial (XL)	5/5	ID	[13]
19	M	0 ^&^	*NDP*	c.295_300del	p.Q99_T100del	Sporadic	5/5	ID	[13]
20	M	0 ^&^	*NDP*	c.88_104del	p.F30Pfs*21	Familial (XL)	5/4	ID	[13]
21	F	8/12	*CTNNB1*	c.283C>T	p.R95*	Familial (de novo)	3/4	MC, ID, paraplegia, p.A475P in *FZD4*	This study
22	F	3	*CTNNB1*	c.1711G>A	p.E571K	Sporadic (de novo)	2/3	MC, ID, cardiovascular abnormalities, bone density loss, hearing loss in right ear, p.G391S in *COL1A2*	This study
23	F	8/12	*CTNNB1*	c.1426delC	p.Q476Kfs*31	Sporadic (de novo)	3/1	MC, ID, spastic paraplegia of lower limbs	This study
24	M	1	*CTNNB1*	c.1949dupG	p.V651Cfs*14	Sporadic (de novo)	1/3	MC, ID, FD, excessive startle, small feet and toe walking	This study
25	F	9/12	*DOCK6*	c.2786_2790dupAGCAC/ c.5154dupT	p.A931Sfs*11/ p.D1719*	Sporadic (AR)	4/4	ID, epilepsy, periventricular calcification, cerebral corpus callosum dysplasia, left 3-finger defect	This study
26	M	4/12	*DOCK6*	c.4849G>A/ c.1292dupC	p.A1617T/ p.R431Pfs*9	Sporadic (AR)	4/4	ID, epilepsy (West syndrome), periventricular calcification	This study
27	M	2	*LRP5*	c.1604C>T/ c.1850T>G	p.T535M/ p.F617C	Sporadic (AR)	4/4	ID, OPPG	[13]
28	M	8/12	*LRP5*	c.433C>T	p.L145F	Familial (AD)	4/4	ID, (affected: mother, brother, sister)	[13]
29	F	0/12	*TSPAN12*	inv(7)(q22q31.3)		Familial	4/2	precocious puberty	This study
30	M	5/12	*TSPAN12*	del(7)(q31.2q32)		Sporadic	1/1	ID, diaphragmatic eventration, pulmonary artery stenosis, toe deformity	This study

^&^ Age of months unknown; AD, autosomal dominant; AR, autosomal recessive; F, female; FD, facial dysmorphism; ID, intellectual disability; LE, left eye; M, male; MC, microcephaly; OPPG, osteoporosis-pseudoglioma syndrome; RE, right eye; XL, X-linked.

The percentages of the more severe eyes in syndromic FEVR were 5% in Stage 1, 2% in Stage 2, 14% in Stage 3, 45% in Stage 4, and 33% in Stage 5. In the non-syndromic FEVR eye, there were 15% in Stage 1, 5% in Stage 2, 15% in Stage 3, 34% in Stage 4, and 14% in Stage 5. The distribution of the stages was significantly different between syndromic and non-syndromic FEVR (*p* = 0.0013). However, when the stages of the more severely affected eyes were compared among patients with the infantile type, no significant difference was observed (median stage, four in both groups; *p* = 0.1932; Table 3). In contrast, the median stage of the less severely affected eyes differed between groups and was more advanced in syndromic FEVR than in non-syndromic FEVR (median, 4 vs. 3; *p* = 0.0001). The most common stage was Stage 4 in both syndromic and non-syndromic FEVR. The more severe eyes at Stage 5 were found more frequently in syndromic FEVR group, while the more severe eyes at Stages 1 to 3 were found more frequently in the non-syndromic FEVR group. Eyes that progressed to rhegmatogenous RD were not observed in the syndromic FEVR eyes but observed in 42 (18%) of the non-syndromic FEVR eyes.

**Table 3:** Demographic characteristics of infantile cases of syndromic and non-syndromic familial exudative vitreoretinopathy (FEVR).

	Syndromic *n* = 40	Non-Syndromic *n* = 136	*p*
Male	21 (53%)	86 (63%)	
Female	19 (48%)	50 (37%)	0.2300
Familial	12 (30%)	62 (46%)	
Sporadic	28 (70%)	74 (54%)	0.0800
Norrin/β variants positive	12 (30%)	84 (62%)	
Norrin/β variants negative	28 (70%)	52 (38%)	0.0005
Asymmetry	12 (30%)	86 (63%)	
Symmetry	28 (70%)	50 (37%)	0.0003
Stage of more severe eyes			
Stage 1	2 (5%)	3 (2%)	
Stage 2	0 (0%)	5 (4%)	
Stage 3	6 (15%)	23 (17%)	
Stage 4	19 (48%)	75 (55%)	
Stage 5	13 (33%)	28 (21%)	
Stage R	0 (0%)	2 (1%)	
Median stage of more severe eyes	4	4	0.1932
Stage of less severe eyes			
Stage 0	0 (0%)	4 (3%)	
Stage 1	7 (18%)	58 (43%)	
Stage 2	3 (8%)	4 (3%)	
Stage 3	4 (10%)	28 (21%)	
Stage 4	16 (40%)	34 (25%)	
Stage 5	10 (25%)	8 (6%)	
Median stage of less severe eyes	4	3	0.0003

### 2.2. FEVR-Associated Variants Found in This Study

Among the 42 syndromic FEVR probands, 71% (30/42) were found to have pathogenic genetic variants or chromosomal rearrangements (Table 2). Twenty probands were reported earlier, and they had pathogenic variants in the Norrin/β-catenin-related genes (*LRP5*, *TSPAN12*, *NDP*) or the *KIF11* gene [13,14]. The variants in seven families were new: three families carried three pathogenic variants in the *CTNNB1* gene (c.1711G>A:p.E571K in Case #22, c.1426delC:p.Q476Kfs*31 in Case #23 and c.1949dupG:p.V651Cfs*14 in Case #24); two probands carried four variants in the *DOCK6* gene compound heterozygously ([c.2786_2790dupAGCAC:p.A931Sfs*11] + [c.5154dupT:p.D1719*] in Case #25 and [c.4849G>A:p.A1617T]+ [c.1292dupC:p.R431Pfs*9] in Case #26). In addition, two families carried two variants of the *KIF11* genes (c.370G>T:p.E124* in Case #12; and c.2842dupC:p.L948Pfs*2 in Case #2). The c.283C>T:p.R95* variant in the *CTNNB1* gene in Case #21 is a reported pathogenic variant associated with intellectual disability, but it was not reported for the FEVR-like phenotype [15]. c.4849G>A:p.A1617T in the *DOCK6* gene was reported to be associated with the phenotype of Adams–Oliver syndrome [16].

The other eight variants were novel variants, and they were not listed in one global and two local population databases (the Genome Aggregation Database [gnomAD], https://gnomad.broadinstitute.org/; the Tohoku Medical Megabank Organization database [Tommo3], https://www.megabank.tohoku.ac.jp/english/; Human Genetic Variation Database [HGVD], https://www.hgvd.genome.med.kyoto-u.ac.jp/). These data were retrieved via the ANNOVAR (https://annovar.openbioinformatics.org/en/latest/) on 6 January 2025, 31 August 2018, and 20 February 2025 for gnomAD, Tommo3, and HGVD, respectively. Based on the ACMG criteria, they were pathogenic or likely pathogenic (Table 4). In addition, two probands had rearrangements on chromosome 7 involving the *TSPAN12* gene: inv(7)(q22q31.3) in Case #29 and del(7)(q31.2q32) in Case #30.

No pathogenic variants in other rarer FEVR-causing genes were identified, including *ZNF408*, *TUBGCP6*, and *JAG1* [17,18,19]. The details of significant unknown variants has been reported [13].

**Table 4:** Novel variants found in this study.

Gene	Nucleotide Change	Protein Change	Pathogenicity: Evidenced Criteria ^a^
*KIF11*	c.370G>T	p.E124*	Pathogenic: PVS1 + PM2 + PS2
*KIF11*	c.2842dupC	p.L948Pfs*2	Likely pathogenic: PVS1 + PM2
*CTNNB1*	c.1426delC	p.Q476Kfs*31	Pathogenic: PVS1 + PM2 + PS2
*CTNNB1*	c.1711G>A	p.E571K	Likely pathogenic: PM2 + PS2 + PP3 ^b^ + PM1 ^c^
*CTNNB1*	c.1949dupG	p.V651Cfs*14	Pathogenic: PVS1 + PM2 + PS2
*DOCK6*	c.5154dupT	p.D1719*	Pathogenic: PVS1 + PM2 + PM3
*DOCK6*	c.1292dupC	p.R431Pfs*9	Pathogenic: PVS1 + PM2 + PM3
*DOCK6*	c.2786_2790dupAGCAC	p.A931Sfs*11	Pathogenic: PVS1 + PM2 + PM3

^a^ Assessments of the pathogenicity of the variants were based on the guideline of the American College of Medical Genetics and Genomics (ACMG) [20]. ^b^ GERP++ (https://bio.tools/gerp) = 5.62; Alphamissense (https://alphamissense.hegelab.org/) = 0.9989; M-CAP (http://bejerano.stanford.edu/mcap/) = 0.092; REVEL (https://genebe.net/hub/@genebe/revel/0.0.1) = 0.598; Polyphen2 (HumDIV, http://genetics.bwh.harvard.edu/pph2/) = 0; CADD (https://cadd.gs.washington.edu/snv) = 35. ^c^ Armadillo (ARM)/beta-catenin-like repeats. (all accessed on 30 March 2026).

### 2.3. Extraocular Phenotypes of Syndromic FEVR with Confirmed Genetic Variants

Of the 30 probands with identified pathogenic variants or chromosomal abnormalities, 20 were sporadic cases, and 11 of these had de novo variants. In all cases, the extraocular signs were present during infancy (Table 2). The systemic signs included intellectual disability in 83% (25/30) probands, microcephaly in 53% (16/30) probands, facial dysmorphism in 27% (8/30) probands, and cardiac abnormalities, epilepsy, periventricular calcification, and paralysis of the lower extremities in 7% (2/30) probands. Osteoporosis, dysplasia of the cerebral corpus callosum, hand defects, decreased bone density, and deafness were also observed in one proband.

There were two cases with chromosomal rearrangements of the *TSPAN12* gene: Case #30 presented with intellectual disability, diaphragmatic eventration, pulmonary artery stenosis, and toe deformity, and Case #29 presented with precocious puberty. Two cases with *LRP5* gene variants presented with intellectual disability, and one of these cases was associated with osteoporosis as a systemic change. This patient was diagnosed with OPPG. All 12 cases with the *KIF11* gene variants presented with microcephaly, and 67% (8/12) also had intellectual disability. All cases with *NDP* gene variants presented with intellectual disability consistent with Norrie disease; however, they did not have the microcephaly phenotype, which is consistently found for patients with *KIF11* mutations.

### 2.4. Genetic and Clinical Characteristics of Patients with Mutations in the CTNNB1 Gene

**Figure 1:**
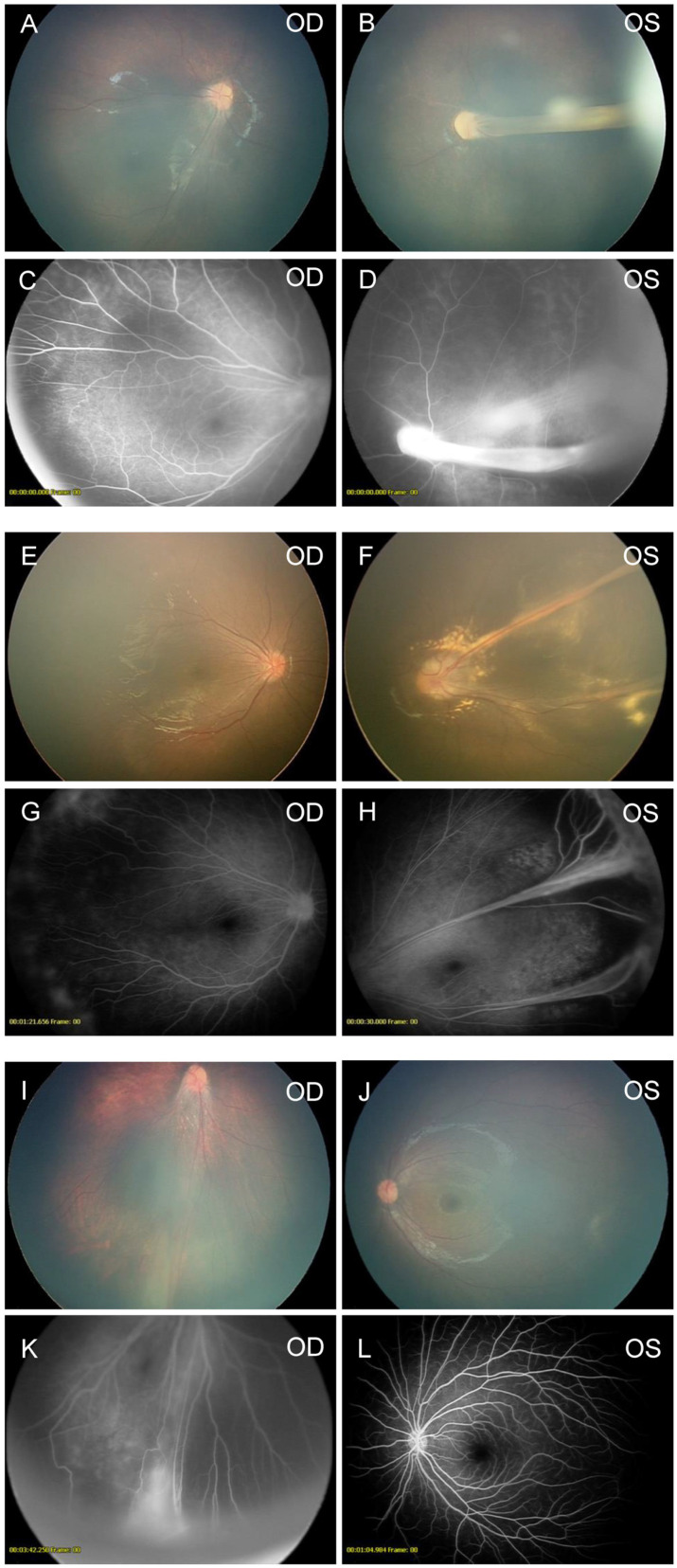
Fundus photographs and fluorescein angiograms of eyes of patients with familial exudative vitreoretinopathy (FEVR) associated with mutations of the *CTNNB1* gene. The right eye and left eye are shown in the left and right panels, respectively. Case #21 (**A**–**D**), Case #22 (**E**–**H**), and Case #23 (**I**–**L**) showing retinal dragging towards the temporal periphery and retinal folds. Note that Case #21 also had a *FZD4* mutation. OD, the right eye; OS, the left eye.

All four cases with a *CTNNB1* gene mutation were de novo (Table 2). Three of the four cases were sporadic; however, the paternal relatives of Case #21 with p.R95* presented with non-syndromic familial FEVR with an additional *FZD4* mutation (p.A475P) [13]. Thus, this proband had digenic mutations in both the *CTNNB1* and *FZD4* genes. Her sister had fundus findings with characteristics of FEVR and intellectual disability, but her genotype has yet to be determined. Case #22 with p.E571K was also complicated by carrying a known disease-associated *COL1A2* variant, c.1171G>A; NM_000089.4:p.G391S (rs67707918, which is likely pathogenic based on the Clinvar database, https://www.ncbi.nlm.nih.gov/clinvar/, accessed on 30 March 2026) with a diagnosis of osteogenesis imperfecta. All patients with *CTNNB1* mutations had microcephaly and intellectual disability, and other systemic changes included motor developmental delay of the lower extremities in Case #21; decreased bone density, deafness in the right ear, and cardiovascular abnormalities in Case #22; spastic paralysis of the lower extremities in Case #23; and mild facial dysmorphism, hyperekplexia, and small feet with toe walking in Case #24. The severity of the FEVR signs of these eight eyes was Stage 4 in one eye (13%), Stage 3 in four eyes (50%), Stage 2 in one eye (13%), Stage 1 in two eyes (25%, Figure 1). None of the eyes had Stage 5 FEVR.

### 2.5. Clinical Characteristics of Patients with Mutations in the DOCK6 Gene

**Figure 2:**
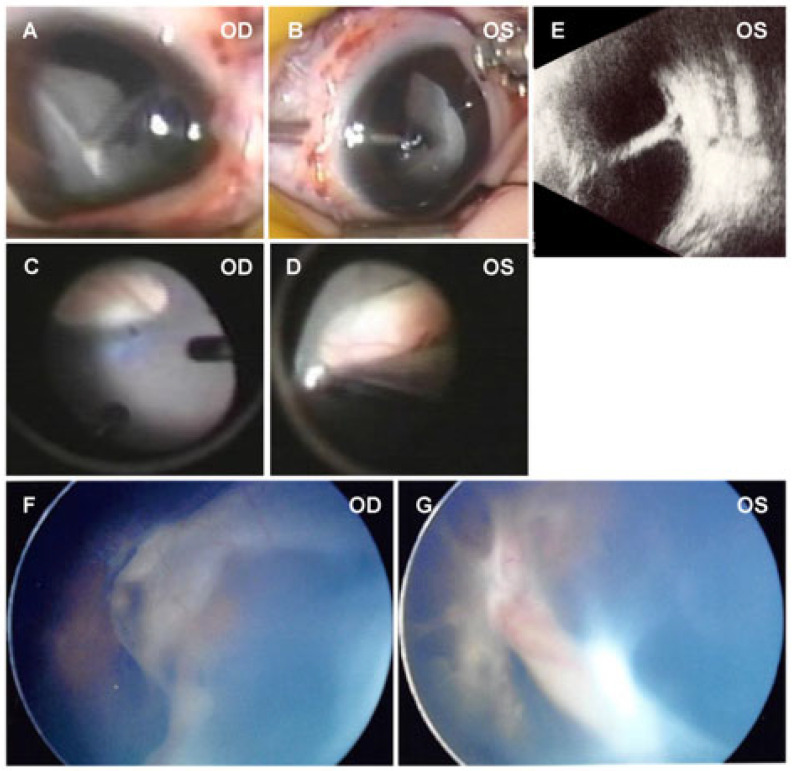
Preoperative and intraoperative fundus photographs of patients with mutations in the *DOCK6* gene. Intraoperative images and ultrasonographic images of Case #25 (**A**–**E**) showing retrolental fibrovascular proliferation in the periphery and dragged disk due to falciform retinal folds. Widefield fundus photographs of Case #26 (**F**,**G**) showing falciform retinal folds. OD, the right eye; OS, the left eye.

**Figure 3:**
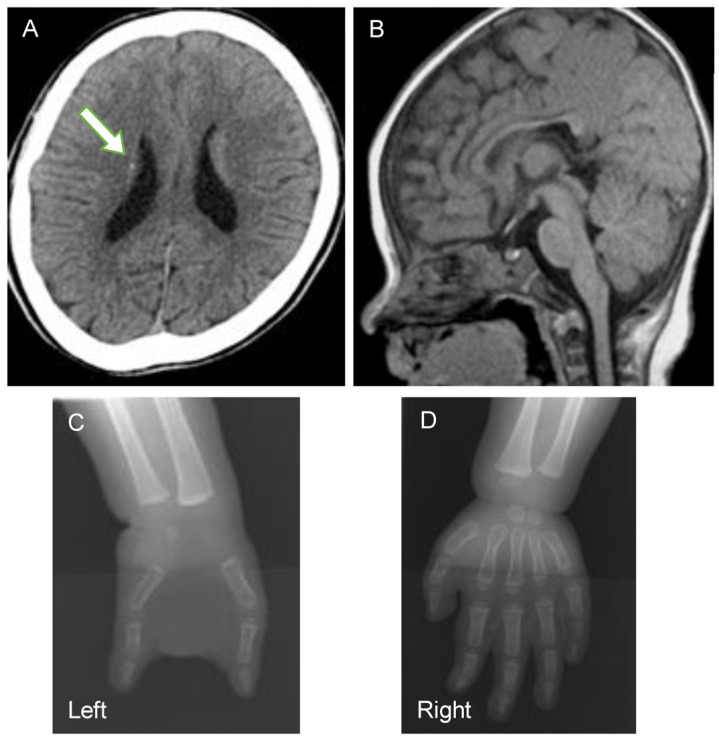
Systemic findings in Case #25 with mutations in the *DOCK6* gene. (**A**): Computed tomographic (CT) image showing periventricular calcification (*arrow*). (**B**): Magnetic Resonance Image showing cerebral corpus callosum dysplasia. (**C**,**D**): X-ray image of the hands; the left hand (**C**) has a three-finger defect while the right hand (**D**) is unaffected.

Case #25 was followed by a pediatrician for intellectual disability and a three-finger defect of the left hand (Figure 2 and Figure 3). At 9 months of age, a tractional RD was found in both eyes, and the patient was referred to the ophthalmology department. A CT scan of the head revealed periventricular calcification, and an MRI scan of the head revealed dysplasia of the corpus callosum and an enlargement of the right ventricle. There were no congenital scalp defects or cardiovascular abnormalities characteristic of Adams–Oliver syndrome. She had Stage 4 FEVR in both eyes with a retrolental fibrous proliferation and falciform retinal folds.

Case #26 had intellectual disability and epilepsy and was diagnosed with the West syndrome and periventricular calcification. He had Stage 4 FEVR with falciform retinal folds in both eyes (Figure 2).

### 2.6. Extraocular Phenotype of Syndromic FEVR Without Confirmed Genetic Variants

Of the 12 probands whose genetic abnormalities were not detected, 75% (9/12) were sporadic cases. Microcephaly was present in 25% (3/12), and intellectual disability in 58% (7/12) probands. Heart disease was detected in 25% (3/12) probands, including two with ventricular septal defect and one with a single ventricle. Finger malformation was noted in two probands. In addition, the following abnormalities were observed in one proband: cerebral bridge defect, renal failure, dental abnormalities, soft laryngeal face, enlarged ventricles, autism, Down syndrome, transient abnormal myeloproliferation, and intracerebral calcification.

## 3. Discussion

Our results showed that patients with syndromic FEVR had genetic and clinical characteristics distinct from patients with non-syndromic FEVR. As has been reported, non-syndromic FEVR cases had (1) large differences in the severity of the FEVR with the presence of Stage 1 to Stage 5, and often asymmetry between the two eyes, (2) cases were diagnosed not only in infancy but also in the adults with late-onset symptoms, and (3) rhegmatogenous RD was present in Japanese patients [13]. On the other hand, syndromic FEVR generally had more severe retinal changes, typically Stage 4 or higher with Stage 1 rarely observed, and they had symmetrical involvement of both eyes. The majority of the syndromic FEVR cases were diagnosed as sporadic cases. The detection rate of Norrin/β-catenin signaling genes (*LRP5*, *FZD4*, and *TSPAN12*) was limited to 29% (12/42).

A link between the pathogenic variants in the *CTNNB1* gene and FEVR-like retinopathy was first reported by Dixon et al. [9]. To date, 24 cases with the *CTNNB1* variants have been reported to have signs of FEVR [8,9,21,22,23,24,25,26,27,28,29]. Except for the two non-syndromic FEVR in three FEVR families reported by Panagiotou et al. [8], all reported families, except for infants without follow-up, had cognitive abnormalities [9,21,22,23,24,25,26,27,28,29]. Therefore, systemic signs are strongly associated with FEVR, as was confirmed in this study, in which the patients consistently presented with microcephaly, facial dysmorphism, or intellectual disabilities. Moreover, unlike the consistently observed cognitive signs in patients with the *CTNNB1* variants, ocular changes including retinopathy were less common (50–75%) in these patients [15,21,30]. The lower frequency of observations of retinal changes contrasts with the findings in patients carrying variants of the other Norrin/β-catenin pathway genes. Earlier studies showed that most cases were Stage 4 or higher [22,27]. In contrast, our study showed that Stage 3 was the most frequently observed (50%) and occurred without ocular asymmetry. This suggested that *CTNNB1* mutations can present with more different retinal severities than previously recognized.

Pathogenic variants of the *DOCK6* gene are known to cause Adams–Oliver syndrome, which is characterized by aplasia cutis congenita, terminal transverse limb defects, and cardiac and vascular anomalies [31]. FEVR-like retinal changes such as tractional retinal detachments have been reported in these patients [32]. Two of the probands with bi-allelic *DOCK6* mutations had signs of Adams–Oliver syndrome. Despite the absence of an initial diagnosis, systemic manifestations including periventricular calcification and finger abnormalities supported this diagnosis, and the cases were diagnosed as syndromic FEVR. Thus, FEVR may be diagnosed in patients with Adams–Oliver syndrome when systemic features are subtle. Moreover, Jin et al. reported a case with mutations of the *DOCK6* gene that lacked any systemic signs but had peripheral retinal nonperfusion, a sign of FEVR [33].

The patients with NDP mutations had two phenotypes, Norrie disease and X-linked FEVR, dependent on the degree of functional loss [6]. Similarly, *LRP5* mutations probably had a reduced bone density phenotype, especially in cases involving dominant-negative or biallelic variants. The associated phenotypes are either FEVR- or OPPG-based on the mutational effects [34]. Although it is rare, microcephaly can be associated with *LRP5* mutations [13,18]. However, an association between microcephaly and *NDP* mutations has not been reported.

Mutations in the *TSPAN12* gene alone have not been associated with systemic abnormalities. However, Seo et al. reported a FEVR patient with a large deletion del(7)(q31.3q33) in the *TSPAN12* gene who presented with congestive heart failure. A large deletion involving the *TSPAN12* gene may cause additional systemic phenotypes that are likely due to the loss of another gene [35].

The genetic causes of syndromic FEVR are hierarchically complex, including (1) more severe variants in the Norrin/β-catenin signaling genes, e.g., *NDP* and *LRP5*; (2) genes different from non-syndromic FEVR, e.g., *KIF11* and *CTNNB1*; (3) overlapping genes which cause a syndrome distinct from FEVR, e.g., *DOCK6*; and (4) incidental associations of other genes independent from the FEVR genes, e.g., *COL1A2* gene in Case #22. In addition, there may be unknown genes for the FEVR phenotype associated with systemic abnormalities.

Future research should focus on collecting a larger number of well-characterized cases to establish a comprehensive understanding of syndromic and non-syndromic FEVR. Further studies are warranted to validate genotype–phenotype associations by identifying causative variants in novel genes.

There are several limitations in this study. First, the number of probands was small, which made it difficult to obtain the complete characteristics of the syndromic FEVR. In addition, not all the mutant genes known to be associated were identified. van der Ende et al. [5] listed additional genes that cause syndromic diseases with a FEVR-like retinal phenotype, e.g., the *NOTCH1*, *ARHGAP31*, *LAMA1*, *COL9A1* and *CDK19* genes. Second, chromosomal testing and MLPA were applied to a limited number of cases which were suspected on the presence of the specific conditions based on their phenotype. A more comprehensive sequence analysis for unsolved cases, i.e., whole genome sequencing, was not used. Third, there is a difficulty in determining the exact frequency of the syndromic FEVR among all FEVR cases. Some systemic signs, including cognitive problems and OPPG-related multiple bone fractures in adolescence, tend to be delayed so that the diagnosis can be undetected with shorter follow-up times. Fourth, the etiology of 29% (12/42) of the syndromic FEVR is yet to be genetically determined. Nonetheless, the strength of this study was that this was the first study focusing on the genetic and phenotypic comparisons between syndromic and non-syndromic FEVR with statistical evaluations.

In conclusion, the genetic characteristics of syndromic FEVR are varied and complex, and these cases have retinal signs distinct from those of non-syndromic FEVR. The information collected on the genotype and phenotype of syndromic FEVR patients should facilitate an earlier diagnosis of FEVR and enhance earlier treatment. For example, early systemic clues may include symmetrical severe retinal detachments with or without microcephaly, which may suggest *KIF11* or *NDP* mutations. These conditions are often associated with subsequent intellectual disability, and early pediatric intervention may be beneficial.

## 4. Materials and Methods

This was a multicenter, retrospective case series study. The procedures used conformed to the tenets of the Declaration of Helsinki, and they were approved by the Ethics Committee of the University Hospital of Occupational and Environmental Health, Japan (Project code 20-148), the Kindai University (22-132), the Jikei University School of Medicine (24-231 6997), and the National Center for Child Health and Development (518). All patients were examined between 2010 and 2023 in the four hospitals. Detailed demographics of the cohorts were reported earlier [13]. Signed informed consent was obtained from all patients or their parents. Patients from the Fukuoka University whose findings were presented in our earlier studies were included and re-evaluated by performing whole exome sequencing (WES) on their DNA samples after approval of the Ethics Committee of Fukuoka University (U21-04-015) [13].

All of the patients were Japanese and were born at full term with normal weight and without a history of either prematurity or oxygen-supplementation (there was one exception; one syndromic case who was born at 34 weeks’ gestation was included). The medical charts of the patients were reviewed, and the ocular images were collected. The diagnosis of FEVR was based on the presence of typical clinical signs, including peripheral retinal avascularization with abnormal vascular development, retinal exudates, neovascularization, peripheral fibrovascular masses, macular ectopia, retinal folds, retinal detachment, and vitreous hemorrhage.

Syndromic FEVR patients were selected from the 281 FEVR probands who underwent genetic testing [13]. Patients who had been diagnosed with other syndromes based on genetic testing prior to the initial ophthalmic examinations were excluded, and one female case with cutis laxa was excluded from the cohort [13]. In addition, one male patient with a chromosome 7 rearrangement involving *TSPAN12*, who had been missing from the previous cohort [13], was newly included in this study. The frequency of each type of systemic disorder and ocular findings was determined.

The ocular examinations included measurements of the refractive error, best-corrected visual acuity, and intraocular pressure. Slit-lamp biomicroscopy, ophthalmoscopy, ultrasonography, optical coherence tomography, fundus photograph and fluorescein angiography were also performed. The details have been reported previously [13]. The severity of FEVR was based on the classification of Pendergast and Trese: Stage 1, avascular peripheral retina; Stage 2, retinal neovascularization; Stage 3, extramacular RD; Stage 4, macula-involving RD; and Stage 5, total RD [36]. In addition, FEVR eyes with rhegmatogenous RD that progressed from Stages 1 or 2 were classified as “R” because these cases had better prognosis than those with rhegmatogenous RD that progressed from more advanced stages [13,37]. The definition of infantile cases was diagnosed at ≤5 years of age with congenital falciform retinal fold or more severe retinopathy in at least one eye. The remaining cases were classified as juvenile or adult patients [13].

The reference sequences of the *FZD4* (NM_012193.4), *NDP* (NM_000266.4), *LRP5* (NM_002335.4), *TSPAN12* (NM_012338.4), *KIF11* (NM_004523.3), *CTNNB1* (NM_001904.4), and *DOCK6* (NM_020812.1) genes were used with a variation number based on their cDNA sequence, with +1 corresponding to the first nucleotide of the initiation codon (ATG). Comprehensive details of the DNA sequence analyses have been given earlier [13]. In brief, DNA samples extracted from the peripheral blood of the probands were screened by Sanger sequencing and/or whole exome sequencing (WES) for their coding sequences. The filtering criteria of the variants for the allele frequency and computational predictions were also described in detail earlier [13]. The assessment of the pathogenicity of the variants was based on the guideline of the American College of Medical Genetics and Genomics (ACMG) [20].

Two patients who were strongly suspected to have genetic variants in the *KIF11* gene were additionally examined by multiple ligation probe assay (MLPA) as reported [14]. Two patients without previously detected mutations underwent conventional karyotype examination due to systemic abnormalities [13].

Statistical analyses were performed with the Prism 9 software (version 9.5.1; GraphPad Software, Boston, MA, USA). Fisher’s exact tests were used for 2 × 2 contingency tables and Chi-square tests for other contingency tables to determine the significance of the categorized data. *p* < 0.05 was taken to be statistically significant.

## Data Availability

The data analyzed in this study are not publicly available because of restrictions related to the protection of personal information and ethical approval, but are available from the corresponding author upon reasonable request.

## Abbreviations

ACMG	American College of Medical Genetics and Genomics
CT	computed tomography
DNA	deoxyribonucleic acid
FEVR	familial exudative vitreoretinopathy
gnomAD	Genome Aggregation Database
HGVD	Human Genetic Variation Database
MLPA	multiple ligation probe assay
MRI	magnetic resonance imaging
OPPG	osteoporosis pseudoglioma
RD	retinal detachment
Tommo3	Tohoku Medical Megabank Organization database
WES	whole exome sequencing
